# (4,5-Diaza­fluoren-9-one-κ^2^ *N*,*N*′)bis­(1*H*-imidazole-κ*N* ^3^)bis­(thio­cyanato-κ*N*)cobalt(II)

**DOI:** 10.1107/S1600536811029060

**Published:** 2011-08-02

**Authors:** Xiu-Ling Feng, Yu-Ping Zhang

**Affiliations:** a College of Chemistry and Chemical Engineering, Huaihua University, Huaihua 418008, People’s Republic of China; b Wuling Electric Power Group Corporation, Changsha 410000, People’s Republic of China

## Abstract

In the title complex, [Co(NCS)_2_(C_3_H_4_N_2_)_2_(C_11_H_6_N_2_O)], the Co^II^ atom has a distorted octa­hedral coordination with the N atoms of the 4,5-diaza­fluoren-9-one ligand and two N atoms from imidazole ligands in the equatorial positions and the axial sites occupied by two N atoms of the thio­cyanate ligand. Inter­molecular N—H⋯O hydrogen bonding forms a one-dimensional motif parallel to the cell *ab* diagonal.

## Related literature

For related structures, see: Notash *et al.* (2011 ▶); Xu *et al.* (2009 ▶). For general background to metal complexes with diazafluoren-9-one ligands, see: Biju & Rajasekharan (2008 ▶); Zhang & Li (2009 ▶). For a related structure, see: Yang *et al.* (2004 ▶).
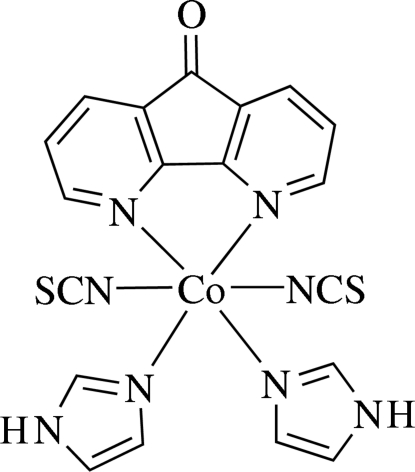


## Experimental

### 

#### Crystal data

[Co(NCS)_2_(C_3_H_4_N_2_)_2_(C_11_H_6_N_2_O)]
*M* *_r_* = 493.43
Triclinic, 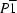

*a* = 9.2239 (9) Å
*b* = 10.920 (1) Å
*c* = 11.9441 (12) Å
α = 71.578 (1)°
β = 70.582 (1)°
γ = 73.931 (2)°
*V* = 1056.48 (18) Å^3^
*Z* = 2
Mo *K*α radiation
μ = 1.04 mm^−1^
*T* = 298 K
0.35 × 0.33 × 0.30 mm

#### Data collection

Bruker SMART CCD area-detector diffractometer
Absorption correction: multi-scan (*SADABS*; Sheldrick, 1996 ▶) *T*_min_ = 0.712, *T*_max_ = 0.746
5357 measured reflections
3633 independent reflections
2208 reflections with *I* > 2σ(*I*)
*R*_int_ = 0.041

#### Refinement

*R*[*F* ^2^ > 2σ(*F* ^2^)] = 0.062
*wR*(*F* ^2^) = 0.192
*S* = 1.06
3633 reflections
280 parameters
H-atom parameters constrained
Δρ_max_ = 1.10 e Å^−3^
Δρ_min_ = −0.50 e Å^−3^

### 

Data collection: *SMART* (Bruker, 2002 ▶); cell refinement: *SAINT* (Bruker, 2002 ▶); data reduction: *SAINT*; program(s) used to solve structure: *SHELXS97* (Sheldrick, 2008 ▶); program(s) used to refine structure: *SHELXL97* (Sheldrick, 2008 ▶); molecular graphics: *SHELXTL* (Sheldrick, 2008 ▶); software used to prepare material for publication: *SHELXTL*.

## Supplementary Material

Crystal structure: contains datablock(s) I, global. DOI: 10.1107/S1600536811029060/qm2016sup1.cif

Structure factors: contains datablock(s) I. DOI: 10.1107/S1600536811029060/qm2016Isup2.hkl

Additional supplementary materials:  crystallographic information; 3D view; checkCIF report

## Figures and Tables

**Table 1:** Hydrogen-bond geometry (Å, °)

*D*—H⋯*A*	*D*—H	H⋯*A*	*D*⋯*A*	*D*—H⋯*A*
N6—H6⋯O1^i^	0.86	2.47	2.980 (10)	119

Symmetry code: (i) 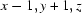.

**Fig. 1.**
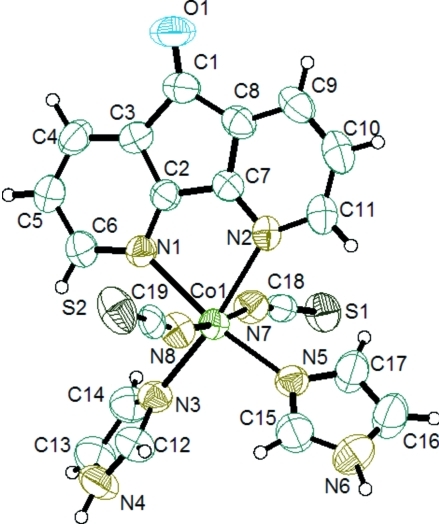
The structure of of the title compound, showing the atomic numbering scheme and 50% probability displacement ellipsoids.

## supplementary crystallographic information

### Comment

The title complex, Co(C_11_H_6_NO_2_)(C_3_H_4_N_2_)_2_(SCN)_2_ contains a Co^II^ centre with a distorted octahedral coordination where the equatorial plane contains the N atoms of 4,5-diazafluoren-9-

one and two N atoms from imidazole ligands and the axial positions are occupied by two N atoms of thiocyanato ligands. Intermolecular N—H···O hydrogen bonding forms a one-dimensional motif parallel to the cell ab diagonal.

### Experimental

A mixture of Co(NO_3_)_2_. 6H_2_O (0.5 mmol), 4,5-diazafluoren-9-one (0.5 mmol), imidazole (0.5 mmol) and KSCN (0.5 mmol) in 15 mL distilled water was heated at 413 K in a Teflon-lined stainless steel autoclave for three days. The reaction system was then slowly cooled to room temperature. Red crystals of the title compound suitable for single-crystal X-ray diffraction analysis were obtained by slow evaporation of the aqueous solution over a period of one month (yield 49% based on Co).

### Refinement

All H atoms attached to C atoms and N atom were fixed geometrically and treated as riding with C—H = 0.93 Å and N—H = 0.86Å with *U*_iso_(H) = 1.2Ueq(C or N).

### Crystal data

**Table:**

[Co(NCS)_2_(C_3_H_4_N_2_)_2_(C_11_H_6_N_2_O)]	*Z* = 2
*M**_r_* = 493.43	*F*(000) = 502
Triclinic, *P*1	*D*_x_ = 1.551 Mg m^−^^3^
Hall symbol: -P 1	Mo *K*α radiation, λ = 0.71073 Å
*a* = 9.2239 (9) Å	Cell parameters from 1602 reflections
*b* = 10.920 (1) Å	θ = 2.4–22.9°
*c* = 11.9441 (12) Å	µ = 1.04 mm^−^^1^
α = 71.578 (1)°	*T* = 298 K
β = 70.582 (1)°	Block, red
γ = 73.931 (2)°	0.35 × 0.33 × 0.30 mm
*V* = 1056.48 (18) Å^3^	

### Data collection

**Table:**

Bruker SMART CCD area-detector diffractometer	3633 independent reflections
Radiation source: fine-focus sealed tube	2208 reflections with *I* > 2σ(*I*)
graphite	*R*_int_ = 0.041
φ and ω scans	θ_max_ = 25.0°, θ_min_ = 2.4°
Absorption correction: multi-scan (*SADABS*; Sheldrick, 1996)	*h* = −10→10
*T*_min_ = 0.712, *T*_max_ = 0.746	*k* = −12→10
5357 measured reflections	*l* = −14→11

### Refinement

**Table:**

Refinement on *F*^2^	Primary atom site location: structure-invariant direct methods
Least-squares matrix: full	Secondary atom site location: difference Fourier map
*R*[*F*^2^ > 2σ(*F*^2^)] = 0.062	Hydrogen site location: inferred from neighbouring sites
*wR*(*F*^2^) = 0.192	H-atom parameters constrained
*S* = 1.06	*w* = 1/[σ^2^(*F*_o_^2^) + (0.0844*P*)^2^ + 0.3689*P*] where *P* = (*F*_o_^2^ + 2*F*_c_^2^)/3
3633 reflections	(Δ/σ)_max_ = 0.001
280 parameters	Δρ_max_ = 1.10 e Å^−^^3^
0 restraints	Δρ_min_ = −0.50 e Å^−^^3^

### Special details

**Table:**

Geometry. All e.s.d.'s (except the e.s.d. in the dihedral angle between two l.s. planes) are estimated using the full covariance matrix. The cell e.s.d.'s are taken into account individually in the estimation of e.s.d.'s in distances, angles and torsion angles; correlations between e.s.d.'s in cell parameters are only used when they are defined by crystal symmetry. An approximate (isotropic) treatment of cell e.s.d.'s is used for estimating e.s.d.'s involving l.s. planes.
Refinement. Refinement of *F*^2^ against ALL reflections. The weighted *R*-factor *wR* and goodness of fit *S* are based on *F*^2^, conventional *R*-factors *R* are based on *F*, with *F* set to zero for negative *F*^2^. The threshold expression of *F*^2^ > σ(*F*^2^) is used only for calculating *R*-factors(gt) *etc*. and is not relevant to the choice of reflections for refinement. *R*-factors based on *F*^2^ are statistically about twice as large as those based on *F*, and *R*- factors based on ALL data will be even larger.

### Fractional atomic coordinates and isotropic or equivalent isotropic displacement parameters (Å^2^)

**Table:**

	*x*	*y*	*z*	*U*_iso_*/*U*_eq_	
Co1	0.20940 (9)	0.77893 (7)	0.26794 (7)	0.0446 (3)	
N1	0.3058 (5)	0.5787 (4)	0.3693 (4)	0.0460 (12)	
N2	0.4108 (6)	0.7047 (5)	0.1105 (4)	0.0478 (12)	
N3	0.0422 (6)	0.8171 (5)	0.4280 (5)	0.0497 (12)	
N4	−0.1818 (7)	0.8644 (6)	0.5558 (6)	0.0788 (18)	
H4	−0.2815	0.8844	0.5855	0.095*	
N5	0.1501 (6)	0.9610 (5)	0.1485 (4)	0.0491 (12)	
N6	0.0312 (10)	1.1390 (7)	0.0524 (7)	0.127 (3)	
H6	−0.0451	1.1975	0.0306	0.152*	
N7	0.3778 (6)	0.8478 (5)	0.2960 (5)	0.0529 (13)	
N8	0.0626 (6)	0.6936 (5)	0.2310 (4)	0.0545 (13)	
O1	0.7400 (6)	0.2887 (5)	0.1886 (5)	0.0799 (15)	
S1	0.5575 (2)	1.00788 (17)	0.30595 (16)	0.0623 (5)	
S2	−0.0800 (3)	0.5262 (2)	0.19374 (19)	0.0811 (6)	
C1	0.6403 (7)	0.3839 (6)	0.2030 (6)	0.0544 (16)	
C2	0.4267 (7)	0.5249 (5)	0.2920 (5)	0.0438 (14)	
C3	0.5196 (7)	0.4031 (6)	0.3187 (6)	0.0504 (15)	
C4	0.4827 (8)	0.3284 (6)	0.4362 (6)	0.0604 (18)	
H4A	0.5395	0.2441	0.4589	0.072*	
C5	0.3613 (8)	0.3810 (6)	0.5177 (6)	0.0618 (17)	
H5	0.3358	0.3333	0.5989	0.074*	
C6	0.2745 (8)	0.5030 (6)	0.4839 (6)	0.0596 (17)	
H6A	0.1901	0.5349	0.5429	0.071*	
C7	0.4808 (6)	0.5879 (5)	0.1624 (5)	0.0428 (13)	
C8	0.6092 (7)	0.5069 (6)	0.1082 (6)	0.0524 (15)	
C9	0.6755 (8)	0.5511 (7)	−0.0145 (6)	0.0619 (18)	
H9	0.7641	0.5015	−0.0564	0.074*	
C10	0.6061 (9)	0.6712 (7)	−0.0729 (6)	0.0664 (19)	
H10	0.6483	0.7043	−0.1561	0.080*	
C11	0.4755 (8)	0.7435 (6)	−0.0107 (6)	0.0569 (17)	
H11	0.4294	0.8231	−0.0543	0.068*	
C12	−0.1081 (8)	0.8433 (7)	0.4456 (6)	0.0650 (18)	
H12	−0.1580	0.8470	0.3883	0.078*	
C13	−0.0723 (11)	0.8488 (8)	0.6116 (7)	0.080 (2)	
H13	−0.0890	0.8560	0.6906	0.096*	
C14	0.0648 (9)	0.8211 (7)	0.5327 (6)	0.0653 (18)	
H14	0.1619	0.8066	0.5472	0.078*	
C15	0.0177 (10)	1.0259 (8)	0.1344 (8)	0.101 (3)	
H15	−0.0766	0.9975	0.1763	0.121*	
C16	0.1769 (11)	1.1482 (8)	0.0101 (7)	0.081 (2)	
H16	0.2211	1.2159	−0.0501	0.097*	
C17	0.2483 (9)	1.0393 (7)	0.0720 (7)	0.073 (2)	
H17	0.3557	1.0192	0.0633	0.088*	
C18	0.4548 (7)	0.9133 (6)	0.2987 (5)	0.0457 (14)	
C19	0.0036 (7)	0.6238 (6)	0.2169 (5)	0.0453 (14)	

### Atomic displacement parameters (Å^2^)

**Table:**

	*U*^11^	*U*^22^	*U*^33^	*U*^12^	*U*^13^	*U*^23^
Co1	0.0439 (5)	0.0449 (5)	0.0449 (5)	−0.0073 (4)	−0.0116 (4)	−0.0120 (4)
N1	0.050 (3)	0.044 (3)	0.041 (3)	−0.006 (2)	−0.012 (2)	−0.009 (2)
N2	0.051 (3)	0.047 (3)	0.044 (3)	−0.017 (2)	−0.010 (2)	−0.007 (2)
N3	0.051 (3)	0.047 (3)	0.053 (3)	−0.008 (2)	−0.011 (2)	−0.020 (2)
N4	0.061 (4)	0.077 (4)	0.083 (5)	−0.012 (3)	0.015 (4)	−0.035 (4)
N5	0.047 (3)	0.049 (3)	0.049 (3)	−0.004 (3)	−0.015 (2)	−0.012 (2)
N6	0.091 (6)	0.097 (6)	0.112 (7)	0.028 (5)	−0.023 (5)	0.036 (5)
N7	0.050 (3)	0.054 (3)	0.058 (3)	−0.010 (3)	−0.019 (3)	−0.013 (3)
N8	0.055 (3)	0.060 (3)	0.052 (3)	−0.015 (3)	−0.013 (3)	−0.017 (3)
O1	0.060 (3)	0.063 (3)	0.109 (4)	0.007 (3)	−0.014 (3)	−0.037 (3)
S1	0.0569 (11)	0.0710 (11)	0.0654 (12)	−0.0240 (9)	−0.0175 (9)	−0.0143 (9)
S2	0.0812 (14)	0.1021 (15)	0.0758 (13)	−0.0518 (12)	−0.0018 (11)	−0.0342 (12)
C1	0.044 (4)	0.053 (4)	0.070 (5)	−0.007 (3)	−0.013 (3)	−0.026 (4)
C2	0.044 (3)	0.043 (3)	0.045 (4)	−0.010 (3)	−0.010 (3)	−0.012 (3)
C3	0.051 (4)	0.045 (3)	0.056 (4)	−0.006 (3)	−0.018 (3)	−0.014 (3)
C4	0.065 (5)	0.045 (4)	0.065 (5)	−0.005 (3)	−0.025 (4)	0.000 (3)
C5	0.070 (5)	0.052 (4)	0.054 (4)	−0.013 (4)	−0.013 (4)	−0.002 (3)
C6	0.065 (4)	0.060 (4)	0.045 (4)	−0.013 (3)	−0.006 (3)	−0.009 (3)
C7	0.043 (3)	0.047 (3)	0.043 (3)	−0.011 (3)	−0.010 (3)	−0.017 (3)
C8	0.043 (4)	0.058 (4)	0.057 (4)	−0.014 (3)	−0.007 (3)	−0.020 (3)
C9	0.056 (4)	0.073 (5)	0.058 (4)	−0.022 (4)	0.005 (3)	−0.032 (4)
C10	0.072 (5)	0.077 (5)	0.053 (4)	−0.037 (4)	−0.002 (4)	−0.015 (4)
C11	0.066 (5)	0.058 (4)	0.048 (4)	−0.026 (3)	−0.012 (3)	−0.008 (3)
C12	0.051 (4)	0.077 (5)	0.066 (5)	−0.009 (4)	−0.010 (4)	−0.027 (4)
C13	0.096 (6)	0.083 (5)	0.057 (5)	−0.014 (5)	−0.003 (5)	−0.033 (4)
C14	0.067 (5)	0.070 (4)	0.063 (5)	−0.006 (4)	−0.020 (4)	−0.026 (4)
C15	0.060 (5)	0.099 (6)	0.090 (6)	0.007 (5)	−0.013 (5)	0.021 (5)
C16	0.089 (6)	0.069 (5)	0.077 (6)	−0.022 (5)	−0.031 (5)	0.008 (4)
C17	0.067 (5)	0.064 (5)	0.081 (5)	−0.021 (4)	−0.027 (4)	0.007 (4)
C18	0.043 (4)	0.051 (4)	0.034 (3)	0.003 (3)	−0.011 (3)	−0.008 (3)
C19	0.051 (4)	0.055 (4)	0.025 (3)	−0.013 (3)	−0.006 (3)	−0.005 (3)

### Geometric parameters (Å, °)

**Table:**

Co1—N7	2.060 (5)	C1—C8	1.487 (9)
Co1—N8	2.062 (5)	C1—C3	1.492 (8)
Co1—N3	2.096 (5)	C2—C3	1.374 (8)
Co1—N5	2.100 (5)	C2—C7	1.457 (8)
Co1—N1	2.237 (4)	C3—C4	1.365 (8)
Co1—N2	2.335 (5)	C4—C5	1.344 (9)
N1—C2	1.318 (7)	C4—H4A	0.9300
N1—C6	1.339 (7)	C5—C6	1.365 (9)
N2—C7	1.319 (7)	C5—H5	0.9300
N2—C11	1.345 (7)	C6—H6A	0.9300
N3—C12	1.293 (8)	C7—C8	1.370 (8)
N3—C14	1.350 (8)	C8—C9	1.369 (8)
N4—C12	1.325 (8)	C9—C10	1.367 (10)
N4—C13	1.332 (9)	C9—H9	0.9300
N4—H4	0.8600	C10—C11	1.370 (9)
N5—C15	1.267 (8)	C10—H10	0.9300
N5—C17	1.331 (8)	C11—H11	0.9300
N6—C16	1.289 (10)	C12—H12	0.9300
N6—C15	1.319 (9)	C13—C14	1.325 (10)
N6—H6	0.8600	C13—H13	0.9300
N7—C18	1.152 (7)	C14—H14	0.9300
N8—C19	1.130 (7)	C15—H15	0.9300
O1—C1	1.197 (7)	C16—C17	1.315 (9)
S1—C18	1.619 (7)	C16—H16	0.9300
S2—C19	1.610 (6)	C17—H17	0.9300
			
N7—Co1—N8	173.1 (2)	C5—C4—C3	117.5 (6)
N7—Co1—N3	93.6 (2)	C5—C4—H4A	121.2
N8—Co1—N3	92.08 (19)	C3—C4—H4A	121.2
N7—Co1—N5	90.24 (19)	C4—C5—C6	121.4 (7)
N8—Co1—N5	92.91 (19)	C4—C5—H5	119.3
N3—Co1—N5	97.45 (19)	C6—C5—H5	119.3
N7—Co1—N1	87.91 (18)	N1—C6—C5	122.8 (6)
N8—Co1—N1	88.00 (19)	N1—C6—H6A	118.6
N3—Co1—N1	91.82 (18)	C5—C6—H6A	118.6
N5—Co1—N1	170.65 (18)	N2—C7—C8	127.8 (6)
N7—Co1—N2	85.39 (18)	N2—C7—C2	122.7 (5)
N8—Co1—N2	88.39 (18)	C8—C7—C2	109.4 (5)
N3—Co1—N2	170.70 (18)	C9—C8—C7	117.2 (6)
N5—Co1—N2	91.80 (18)	C9—C8—C1	134.9 (6)
N1—Co1—N2	78.92 (17)	C7—C8—C1	107.9 (5)
C2—N1—C6	114.3 (5)	C10—C9—C8	117.2 (6)
C2—N1—Co1	108.3 (4)	C10—C9—H9	121.4
C6—N1—Co1	137.4 (4)	C8—C9—H9	121.4
C7—N2—C11	113.5 (5)	C9—C10—C11	121.1 (7)
C7—N2—Co1	105.9 (4)	C9—C10—H10	119.4
C11—N2—Co1	140.5 (4)	C11—C10—H10	119.4
C12—N3—C14	105.3 (6)	N2—C11—C10	123.1 (6)
C12—N3—Co1	125.9 (4)	N2—C11—H11	118.5
C14—N3—Co1	128.8 (4)	C10—C11—H11	118.5
C12—N4—C13	107.0 (6)	N3—C12—N4	111.4 (6)
C12—N4—H4	126.5	N3—C12—H12	124.3
C13—N4—H4	126.5	N4—C12—H12	124.3
C15—N5—C17	103.3 (6)	C14—C13—N4	106.7 (7)
C15—N5—Co1	130.3 (5)	C14—C13—H13	126.6
C17—N5—Co1	126.3 (4)	N4—C13—H13	126.6
C16—N6—C15	109.3 (7)	C13—C14—N3	109.6 (7)
C16—N6—H6	125.3	C13—C14—H14	125.2
C15—N6—H6	125.3	N3—C14—H14	125.2
C18—N7—Co1	164.4 (5)	N5—C15—N6	111.0 (8)
C19—N8—Co1	165.8 (5)	N5—C15—H15	124.5
O1—C1—C8	127.0 (6)	N6—C15—H15	124.5
O1—C1—C3	127.4 (6)	N6—C16—C17	103.7 (7)
C8—C1—C3	105.6 (5)	N6—C16—H16	128.2
N1—C2—C3	126.5 (6)	C17—C16—H16	128.2
N1—C2—C7	124.0 (5)	C16—C17—N5	112.6 (7)
C3—C2—C7	109.5 (5)	C16—C17—H17	123.7
C4—C3—C2	117.5 (6)	N5—C17—H17	123.7
C4—C3—C1	135.0 (6)	N7—C18—S1	177.9 (6)
C2—C3—C1	107.5 (6)	N8—C19—S2	178.7 (5)
			
N7—Co1—N1—C2	−83.4 (4)	C7—C2—C3—C1	0.1 (6)
N8—Co1—N1—C2	91.1 (4)	O1—C1—C3—C4	−0.2 (12)
N3—Co1—N1—C2	−176.9 (4)	C8—C1—C3—C4	178.7 (7)
N2—Co1—N1—C2	2.3 (4)	O1—C1—C3—C2	−178.1 (6)
N7—Co1—N1—C6	95.8 (6)	C8—C1—C3—C2	0.8 (6)
N8—Co1—N1—C6	−89.7 (6)	C2—C3—C4—C5	−2.0 (9)
N3—Co1—N1—C6	2.3 (6)	C1—C3—C4—C5	−179.7 (6)
N2—Co1—N1—C6	−178.5 (6)	C3—C4—C5—C6	2.1 (10)
N7—Co1—N2—C7	86.0 (4)	C2—N1—C6—C5	0.4 (9)
N8—Co1—N2—C7	−91.1 (4)	Co1—N1—C6—C5	−178.7 (4)
N5—Co1—N2—C7	176.1 (4)	C4—C5—C6—N1	−1.4 (11)
N1—Co1—N2—C7	−2.8 (3)	C11—N2—C7—C8	1.4 (8)
N7—Co1—N2—C11	−92.8 (6)	Co1—N2—C7—C8	−177.7 (5)
N8—Co1—N2—C11	90.2 (6)	C11—N2—C7—C2	−177.9 (5)
N5—Co1—N2—C11	−2.7 (6)	Co1—N2—C7—C2	3.0 (6)
N1—Co1—N2—C11	178.5 (6)	N1—C2—C7—N2	−1.1 (9)
N7—Co1—N3—C12	151.9 (5)	C3—C2—C7—N2	178.3 (5)
N8—Co1—N3—C12	−32.0 (6)	N1—C2—C7—C8	179.5 (5)
N5—Co1—N3—C12	61.2 (6)	C3—C2—C7—C8	−1.1 (7)
N1—Co1—N3—C12	−120.1 (5)	N2—C7—C8—C9	0.6 (9)
N7—Co1—N3—C14	−27.4 (6)	C2—C7—C8—C9	−180.0 (5)
N8—Co1—N3—C14	148.7 (5)	N2—C7—C8—C1	−177.8 (5)
N5—Co1—N3—C14	−118.1 (5)	C2—C7—C8—C1	1.6 (6)
N1—Co1—N3—C14	60.6 (5)	O1—C1—C8—C9	−0.6 (12)
N7—Co1—N5—C15	−141.3 (7)	C3—C1—C8—C9	−179.5 (7)
N8—Co1—N5—C15	44.9 (7)	O1—C1—C8—C7	177.4 (6)
N3—Co1—N5—C15	−47.6 (7)	C3—C1—C8—C7	−1.5 (6)
N2—Co1—N5—C15	133.3 (7)	C7—C8—C9—C10	−1.3 (9)
N7—Co1—N5—C17	35.0 (6)	C1—C8—C9—C10	176.6 (6)
N8—Co1—N5—C17	−138.8 (6)	C8—C9—C10—C11	−0.2 (10)
N3—Co1—N5—C17	128.7 (6)	C7—N2—C11—C10	−2.9 (8)
N2—Co1—N5—C17	−50.4 (6)	Co1—N2—C11—C10	175.8 (4)
N3—Co1—N7—C18	−84.9 (19)	C9—C10—C11—N2	2.5 (10)
N5—Co1—N7—C18	12.5 (19)	C14—N3—C12—N4	0.3 (8)
N1—Co1—N7—C18	−176.6 (19)	Co1—N3—C12—N4	−179.2 (4)
N2—Co1—N7—C18	104.3 (19)	C13—N4—C12—N3	−0.9 (9)
N3—Co1—N8—C19	−110 (2)	C12—N4—C13—C14	1.1 (9)
N5—Co1—N8—C19	153 (2)	N4—C13—C14—N3	−1.0 (9)
N1—Co1—N8—C19	−18 (2)	C12—N3—C14—C13	0.5 (8)
N2—Co1—N8—C19	61 (2)	Co1—N3—C14—C13	179.9 (5)
C6—N1—C2—C3	−0.4 (8)	C17—N5—C15—N6	0.5 (10)
Co1—N1—C2—C3	179.0 (5)	Co1—N5—C15—N6	177.5 (6)
C6—N1—C2—C7	178.9 (5)	C16—N6—C15—N5	0.9 (12)
Co1—N1—C2—C7	−1.7 (6)	C15—N6—C16—C17	−1.9 (11)
N1—C2—C3—C4	1.2 (9)	N6—C16—C17—N5	2.3 (10)
C7—C2—C3—C4	−178.2 (5)	C15—N5—C17—C16	−1.8 (9)
N1—C2—C3—C1	179.5 (5)	Co1—N5—C17—C16	−178.9 (5)

### Hydrogen-bond geometry (Å, °)

**Table:**

*D*—H···*A*	*D*—H	H···*A*	*D*···*A*	*D*—H···*A*
N6—H6···O1^i^	0.86	2.47	2.980 (10)	119

Symmetry codes: (i) *x*−1, *y*+1, *z*.
